# High Phenotypic Variation between an In Vitro-Passaged Fowl Adenovirus Serotype 1 (FAdV-1) and Its Virulent Progenitor Strain despite Almost Complete Sequence Identity of the Whole Genomes

**DOI:** 10.3390/v14020358

**Published:** 2022-02-09

**Authors:** Beatrice Grafl, Anna Schachner, Michael Hess

**Affiliations:** 1 Clinic for Poultry and Fish Medicine, Department for Farm Animals and Veterinary Public Health, University of Veterinary Medicine (Vetmeduni Vienna), 1210 Vienna, Austria; michael.hess@vetmeduni.ac.at; 2 Christian Doppler Laboratory for Innovative Poultry Vaccines (IPOV), University of Veterinary Medicine, 1210 Vienna, Austria; anna.schachner@vetmeduni.ac.at

**Keywords:** poultry, fowl adenovirus, gizzard erosion, attenuation, genome

## Abstract

Adenoviral gizzard erosion is an emerging disease with negative impact on health and production of chickens. In this study, we compared in vitro and in vivo characteristics of a fowl adenovirus serotype 1 (FAdV-1), attenuated by 53 consecutive passages in primary chicken embryo liver (CEL) cell cultures (11/7127-AT), with the virulent strain (11/7127-VT). Whole genome analysis revealed near-complete sequence identity between the strains. However, a length polymorphism in a non-coding adenine repeat sequence (11/7127-AT: 11 instead of 9) immediately downstream of the hexon open reading frame was revealed. One-step growth kinetics showed delayed multiplication of 11/7127-AT together with significantly lower titers in cell culture (up to 4 log_10_ difference), indicating reduced replication efficiency in vitro. In vivo pathogenicity and immunogenicity were determined in day-old specific pathogen-free layer chicks inoculated orally with the respective viruses. In contrast to birds infected with 11/7127-VT, birds infected with 11/7127-AT did not exhibit body weight loss or severe pathological lesions in the gizzard. Virus detection rates, viral load in organs and virus excretion were significantly lower in birds inoculated with 11/7127-AT. Throughout the experimental period, these birds did not develop measurable neutralizing antibodies, prevalent in birds in response to 11/7127-VT infection. Differences in pathogenicity between the virulent FAdV-1 and the attenuated strain could not be correlated to prominently discriminate genomic features. We conclude that differential in vitro growth profiles indicate that attenuation is linked to modulation of viral replication during interaction of the virus with the host cells. Thus, hosts would be unable to prevent the rapid replication of virulent FAdV leading to severe tissue damage, a phenomenon broadly applicable to further FAdV serotypes, considering the substantial intra-serotype virulence differences of FAdVs and the variation of diseases.

## 1. Introduction

Fowl adenoviruses (FAdVs) are non-enveloped, dsDNA viruses classified into the family *Adenoviridae*, genus *Aviadenovirus* [1]. To date, five species (*Fowl aviadenovirus* A to *Fowl aviadenovirus* E, FAdV-A to FAdV-E) are recognized, based mainly on molecular criteria of sequencing data, with 12 subordinate serotypes (FAdV-1 to -8a and -8b to -11) defined by cross-neutralization tests [1,2]. In chickens, FAdVs are considered ubiquitous and may be isolated from clinically healthy poultry flocks with single or mixed infections of different FAdVs [3,4,5,6]. However, different serotypes and even strains of the same FAdV serotype differ in their ability to produce illness and mortality [7,8,9]. Thereby, comprehensive epidemiological investigations together with experimental studies to reproduce lesions have defined certain FAdV species/serotypes as primary pathogens of the following diseases: adenoviral gizzard erosion (AGE), hepatitis-hydropericardium syndrome (HHS) and inclusion body hepatitis (IBH) [10,11].

Outbreaks of AGE can affect meat- and layer-type chickens. In young broilers, pullets and laying hens reduced performance (e.g., growth retardation, decreased egg production) and/or higher mortality rates have been documented during clinical outbreaks of the disease [12,13,14,15,16,17]. Economic losses due to gizzard condemnations resulting from subclinical FAdV infections were documented in broilers in the course of slaughterhouse inspections [18,19,20]. Thus far, the disease has been reported from Europe, Middle East and Asia [21]. In the majority of reports, FAdV-1 was defined as the etiological agent of AGE and clinicopathological signs of AGE were successfully reproduced in specific-pathogen-free (SPF) layers and broilers using virulent FAdV-1 field isolates [10,21]. Transmission of FAdVs can occur by the horizontal or vertical route [10]. The widespread nature of the disease and its negative impact on poultry health, welfare and production indicate the need for safe and efficacious protection strategies.

The basis for the control of several viral poultry diseases comprises the development of live attenuated vaccines. Classically, attenuated vaccines are derived from virulent strains by continuous passage in susceptible host-laboratory systems, a product of natural genetic variability and/or induced adaptive mutations [22]. Embryonated chicken eggs or cell cultures are commonly used for isolation and propagation of FAdVs [10]. Adaptation of FAdV-4 to quail fibroblast cells (QT-35) or consecutive passages of the virus in chicken embryos were shown to attenuate the virus [23,24]. Similarly, consecutive passage of FAdV-8b in primary chicken embryo liver (CEL) cells resulted in reduced virus infectivity in cell culture and pathogenicity in SPF chickens [25]. Molecular investigations of FAdV strain variants and genetic markers of virulence are ongoing [26,27,28,29]. Additionally, the recent discovery of recombinant FAdVs merits special attention with regard to identification of virulence factors [30,31]. So far, it remains to be determined whether single amino acid differences noticed in fiber genes of FAdV-1, deletions in the fiber-2 of FAdV-4 or the recently described single mutation introduced into the hexon gene of FAdV-4 can be transferred to other serotypes [31,32,33]. The impact of long-term, consecutive in vitro passaging on genetic changes and replication fitness on fowl adenoviruses has so far hardly been investigated with no data from FAdV-1.

The aim of this study was to elucidate phenotypic variations in context with genomic changes of a FAdV-1 strain pair, obtained from the same isolate at different passage levels in primary CEL cells. For this, we compared in vitro and in vivo properties of the isolate after long-term, consecutive passage to those of the virulent, progenitor strain. Viral growth kinetics were assessed and clinicopathological changes and immunogenicity were investigated in day-old SPF layers. Furthermore, whole genome analysis was performed with consecutive bioinformatics analysis.

## 2. Materials and Methods

### 2.1. Preparation of Primary Chicken Embryo Liver (CEL) Cells

For virus isolation, attenuation and propagation, CEL cell cultures were prepared from livers of 13–15 day-old SPF chicken embryos (VALO Biomedia GmbH, Osterholz-Scharmbeck, Germany) according to a protocol from Schat and Sellers [34]. Such cells were also used for virus titration and to establish one-step growth-curves. CEL cells were grown to near confluence in M199 Medium (Invitrogen/Gibco, Paisley, UK) with 10% of fetal bovine serum (FBS), 10% tryptose phosphate and 0.5% of antibiotics solution (all Invitrogen/Gibco) in a controlled atmosphere of 5% CO_2_ at 37 °C. After infection with the virus and 60–90 min for virus adsorption, the cells were maintained with M199 medium containing all the additions, except that FBS was reduced to 2%.

### 2.2. Virus Origin and Preparation

The FAdV-1 strain was obtained from an AGE outbreak in a commercial broiler flock with clinical signs including weight retardation and increased mortality [13]. The virulent virus was isolated from pooled gizzard samples, plaque purified and labeled at the third passage as 11/7127-VT. It was demonstrated to be pathogenic, capable of inducing gizzard erosions following infection of broiler chickens up to 21 days of age [35,36]. The virus was propagated and passaged continuously for approximately a year in weekly intervals on CEL cell cultures and passage 53 (11/7127-AT) was then used for further investigations. Viral titers were determined by end-point titration [37]. The absence of selected avian pathogens (avian reovirus, infectious bursal disease virus and chicken infectious anemia virus) was confirmed in both virus preparations using in-house established PCR methods.

### 2.3. Virus Replication Kinetics In Vitro

One-step growth curves of 11/7127-VT and -AT, respectively, were determined as described by Alexander et al. [38]. For this, CEL cells were inoculated with the respective virus at a multiplicity of infection of five. After adsorption for 1 h at 37 °C, fresh maintenance medium was added and infected cells were incubated at 37 °C and 5% CO_2_. Both, cell culture supernatant and cells were harvested in 6 h intervals until the majority of the monolayer showed an extensive cytopathic effect (CPE) and cells detached from the flask. To determine extracellular virus, cell culture medium was collected at each time point and frozen at −40 °C. Subsequently, the CEL monolayer was washed three times with sterile phosphate buffered saline (PBS) (Gibco/Thermo Scientific, Vienna, Austria), 1 mL of medium was placed on the monolayer followed by three freeze-thaw cycles before collecting the medium to assess intracellular virus yield. Growth curves were established in duplicate. Titers of infection material (0 h) along with titers of intracellular and extracellular virus post infection (PI) were determined by end-point titration and expressed as tissue culture infectious dose per mL (TCID_50_/mL).

### 2.4. Whole Genome Sequencing and Analysis

Previously, the complete genome sequence of 11/7127-VT was determined and compared to other pathogenic and non-pathogenic FAdV-1 strains [30]; the sequence is deposited in GenBank under accession number MK572848. From 11/7127-AT cell culture supernatant DNA preparation, whole-genome sequencing using an Illumina system (MiSeq V3, Central Service Facility NGS Unit, Vienna, Austria) as well as genome assembly and analyses were performed according to the protocol of Schachner et al. [30]. In addition, regions of interest were amplified by PCR and sequenced to complete illumina data. Furthermore, a rightmost genomic sequence portion of 4484 bp length in 11/7127-AT, for which sufficient read data could not be generated, was completed by Sanger sequencing, using 6 primer pairs with overlapping binding sites (Table S1).

### 2.5. In Vivo Study—Experimental Design and Sampling

Embryonated SPF layer eggs were obtained from VALO Biomedia GmbH (Osterholz-Scharmbeck, Germany) and incubated at our facility. Hatched chicks were individually marked by SwiftackTM (Heartland Animal Health, Inc., Fair Play, MO, USA) and allocated randomly into three groups (group 11/7127-VT and 11/7127-AT with 25 chicks, each, and group NC with 15 chicks). Throughout the experimental period, groups were kept in separate isolator units under negative pressure (Montair Environmental Solutions B.V., Kroneberg, The Netherlands). Birds were provided feed and water *ad libitum*.

In accordance with an established infection model [35], day-old SPF chicks of experimental groups 11/7127-VT and 11/7127-AT were infected via a crop tube placed on a syringe (Omnifix F Solo 1 mL; B. Braun Austria GmbH, Maria Enzersdorf, Austria) with 0.5 mL of 10^6.0^ TCID_50_/mL of the respective virus. Chicks in group NC were kept as negative controls and received sterile PBS. Throughout the experiment, birds were examined daily for clinical signs and body weights were measured in intervals of 3 to 4 days. Cloacal swabs were collected at 0, 3, 7, 10, 14 and 17 days post infection (DPI). Blood samples were collected in weekly intervals from the wing vein and during euthanasia after intravenous application of thiopental (Sandoz, Kundl, Austria) at 3, 7, 10, 14 and 17 DPI (5 birds from group 11/7127-VT and 11/7127-AT, 3 birds from group NC, respectively) from the right jugular vein. Routine post-mortem investigations were performed and pathological changes of the gizzard were evaluated according to an established scoring system with lesion scores (LS) of 0 (normal, no changes), 1 (mild lesions), 2 (moderate lesions) and 3 (severe lesions). Gizzard koilin layer and mucosa were assessed separately [35]. Tissue samples of gizzards and livers were collected to assess histopathological changes and/or viral load.

The animal experiment was discussed and approved by the institutional ethics committee and licensed by the national authority according to §26 of the Law for Animal Experiments, Tierversuchsgesetz 2012—BGBl. I Nr. 114/2012 (license number BMBWF-68.205/0223-V/3b/2018).

### 2.6. Histology

Following post-mortem investigations, gizzard samples were collected, fixed in 3.5% neutral buffered formalin and then embedded in paraffin blocks. From the paraffin-embedded gizzards, 3 µm thick tissue slices were prepared using a Microm HM 360 microtome (Microm Laborgeräte GmbH, Walldorf, Germany). They were mounted on glass slides and stained with haematoxylin and eosin.

### 2.7. Virus Isolation and Quantitative PCR (qPCR)

Gizzard, liver and cloacal swab samples collected throughout the in vivo study were stored at −20 °C until processing. Organ homogenates (20 v%) were prepared with PBS containing 1 mg/mL streptomycin and 100,000 IU/mL penicillin using a T 25 digital ULTRA-TURRAX^®^ (IKA, Staufen, Germany). Cloacal swabs were placed in 1 mL of the same PBS/antibiotics solution. Samples were filter sterilized using syringe filters with a pore size of 0.2 μm (VWR, Vienna, Austria). For virus isolation, nearly confluent CEL cells prepared on 48-well plates (Sarstedt GesmbH, Wiener Neudorf, Austria) were inoculated with 100 μL of sample material. The cultures were incubated and observed for up to five days post infection or until a CPE was detected. Samples were considered negative when no CPE was noticed during three passages.

From the same samples, DNA extraction was performed using the DNeasy Blood and Tissue Kit (Qiagen, Hilden, Germany) according to the manufacturer’s instructions. Extraction controls (i.e., 180 μL sterile PBS in place of sample material) were extracted alongside organ and cloacal swab samples to detect any environmental or laboratory contamination. In order to detect and quantify virus DNA in sample material (organ homogenates and cloacal swab/PBS—antibiotics mix), a SYBR green-based qPCR targeting highly conserved regions in the 52k and pIIIa genes was performed according to Günes et al. [39]. All samples were amplified in duplicate. Extraction controls and negative controls without template were included in all qPCR runs. Fluorescence data was recorded and analyzed on the Rotor-Gene Q thermal cycler (Qiagen, Hilden, Germany) controlled by the Rotor-Gene Q software 1.7 (Qiagen, Hilden, Germany). The number of copies of FAdV DNA per reaction mixture was calculated by comparing threshold cycle values of investigated samples with a well-defined standard curve [39]. The specificity of the 176 bp amplification product was confirmed by melting curve analysis and agarose gel electrophoresis.

### 2.8. Detection of Virus Neutralizing Antibodies

Serum samples obtained during the in vivo study were stored at −20 °C until processing. Samples were inactivated for 30 min at 56 °C and tested for virus neutralizing antibodies against FAdV-1 (reference strain CELO). The virus neutralization test (VNT) was performed in CEL cells on 96-well plates (Sarstedt, Wiener Neudorf, Austria) according to a constant virus-diluted serum method using 100 TCID_50_ per 100 µL inoculum. Plates were incubated and evaluated after 5 days. An antibody titer below or equal to 3 log_2_ was regarded as negative.

### 2.9. Statistics

Initial analysis of the datasets was carried out using the Shapiro–Wilk test associated with a visual inspection of histograms and normal Q–Q plots in order to assess the normal distribution assumptions. Mean body weights as well as viral load/titers from samples of in vivo and in vitro studies were compared via Student’s t-test. Pairwise comparisons for datasets not meeting the normality assumptions (gizzard lesion scores) were carried out with the Mann–Whitney U test (MWU). In each case, *p* values  ≤  0.05 were considered statistically significant. Statistical analyses were performed with the software package SPSS Version 26 (IBM SPSS Statistics; IBM Corp., Armonk, NY, USA).

## 3. Results

### 3.1. Virus Replication In Vitro

**Figure 1:**
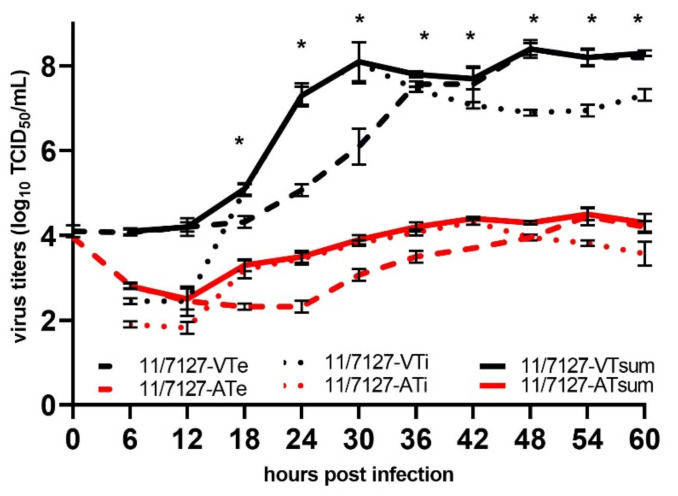
One step growth curve of 11/7127-VT and 11/7127-AT in CEL cells. Samples of both intracellular (11/7127-VTi and -ATi) and extracellular virus (11/7127-VTe and -ATe) were taken at 6 h intervals until 60 h post infection and summarized for total virus yield (11/7127-VTsum and -ATsum). Virus titers were determined by end-point titration. Data shown are from experiments performed in duplicate, with error bars indicating standard deviations. Asterisks indicate a significant difference of the total virus yield between 11/7127-VT and 11/7127-AT.

Both viruses showed similar CPE, typical for fowl adenoviruses. However, with 11/7127-VT, a CPE characterized by focal swelling and detaching of cells was detectable from 30 h PI onwards, while the CPE following infection of cells with 11/7127-AT appeared slightly delayed at 36 h PI. Results of the growth curves in CEL cells are illustrated in Figure 1. Extra- and intracellular 11/7127-VT virus was detected from 12 h PI onward. Titers showed fast, exponential increase. Total virus yields of 11/7127-VT were above 10^7.5^ TCID_50_/mL from 30 h PI onwards and peaked at 48 h PI with 10^8.4^ TCID_50_/mL. Intracellular virus production of 11/7127-AT began between 12 and 18 h PI. Extracellular virus production was first noticed at 24 h PI with titers exceeding intracellular virus after 48 h PI. Total virus yield of 11/7127-AT reached titers above 10^4.0^ TCID_50_/mL from 36 h PI onwards with the maximum virus yield of 10^4.5^ TCID_50_/mL being obtained at 54 h PI. From 18 h onwards, total virus yield of 11/7127-AT remained significantly lower compared to 11/7127-VT (*p* < 0.05) (Figure 1).

### 3.2. Molecular Analysis and Whole Genome Sequence Comparison

The complete genome sequence of the progenitor strain 11/7127-VT has been deposited in GenBank, under accession number MK572848 by Schachner et al. [30]. The genome size is 43,940 base pairs (bp) with a G + C content of 54.3%. From 11/7127-AT, a near-complete genome sequence with 43,795 bp length was obtained with the sequence incomplete in the non-coding, right-terminal region. The obtained sequence showed nearly 100% sequence identity with the progenitor strain; the only identified change between the two genomes was a length polymorphism in a non-coding adenine repeat sequence immediately downstream of the hexon open reading frame, where 11/7127-AT featured a series of eleven, instead of the nine adenines in 11/7127-VT.

### 3.3. In Vivo Study—Clinical Signs and Lesions

**Figure 2:**
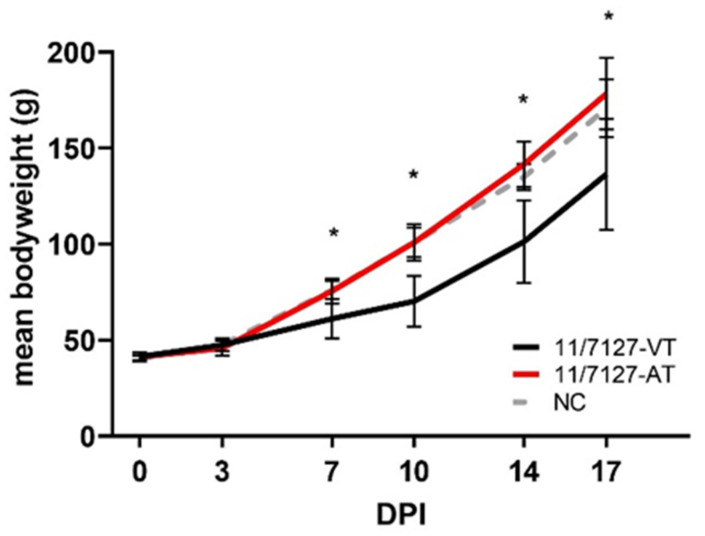
Graphical illustration of mean body weight. Mean body weight (g) from experimentally infected groups 11/7127-VT, 11/7127-AT and from the negative control group (NC) at intervals of 3 to 4 days post infection (DPI). Error bars indicate standard deviations and asterisks a significant difference between group 11/7127-VT and group 11/7127-AT.

Birds inoculated with 11/7127-VT showed a significantly decreased body weight compared to those in group 11/7127-AT from 7 DPI onwards and to the uninfected NC birds at 7, 10 and 14 DPI (*p* < 0.05) (Figure 2). No significant differences in weight gain could be detected between birds of group 11/7127-AT and group NC throughout the trial. No other clinical signs were observed throughout the study.

**Figure 3:**
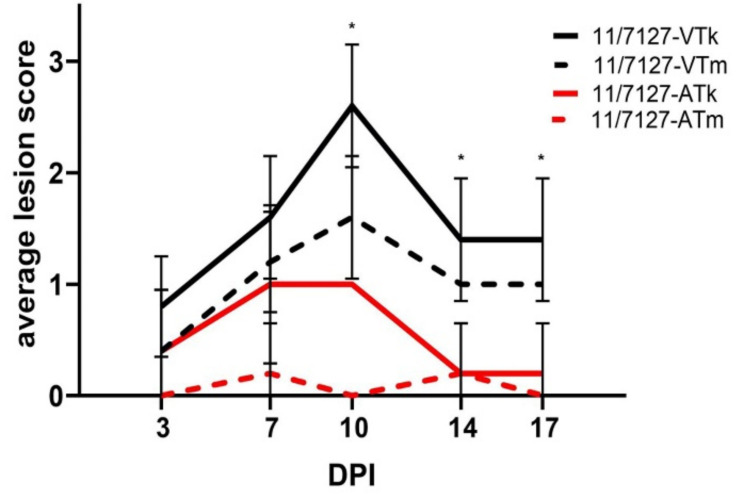
Graphical illustration of gizzard lesion scores. Average lesion scores of gizzard koilin layer (11/7127-VTk and -ATk) and of mucosal membrane (11/7127-VTm and -ATm) at 3, 7, 10, 14 and 17 days post infection (DPI) from SPF layers orally infected with 11/7127-VT or 11/7127-AT. Lesion scores were defined from 0 (no lesions) to 3 (severe lesions). Error bars indicate standard deviations and asterisks indicate a significant difference of gizzard lesions between group 11/7127-VT and group 11/7127-AT.

Average LS detected in gizzard koilin layer and mucosa throughout the experimental investigations are illustrated in Figure 3. In group 11/7127-VT, four and two out of five birds showed lesions of the koilin layer and the gizzard mucosa, respectively, at 3 DPI. From 7 DPI onwards, macroscopic lesions were observed in all of the remaining birds. The most severe macroscopic lesions of both koilin layer and mucosa were observed at 10 DPI, with an average LS of 2.6 and 1.6, respectively. In group 11/7127-AT, koilin layers showed lesions in all birds at 7 and 10 DPI (average LS: 1.0). In addition, lesions of the koilin layer were observed in two, one and one out of five birds at 3, 14 and 17 DPI, respectively. However, gross lesions of the mucosa were detected only sporadically. Thus, while gross lesions of the koilin layer were observed in both groups throughout the trial, average LS of the koilin layer in group 11/7127-AT were lower compared to group 11/7127-VT throughout the experimental period; significant differences between the groups were seen at 10, 14 and 17 DPI (*p* < 0.05). Likewise, average LS of the gizzard mucosa were significantly lower in birds infected with 11/7127-AT in comparison to birds from group 11/7127-VT from 7 DPI onwards (*p* < 0.05). No gross pathological changes were observed in other organs of the birds.

**Figure 4:**
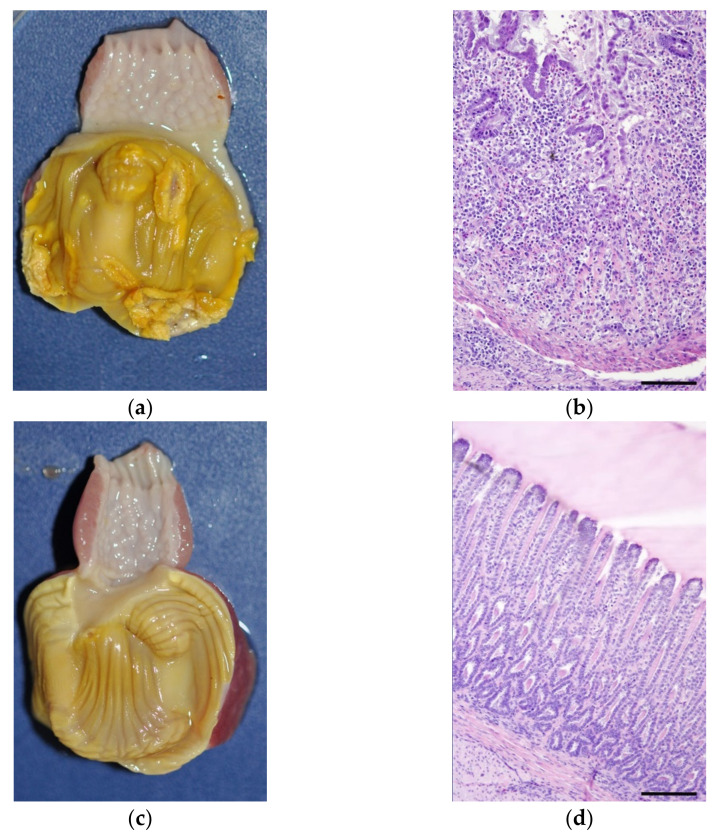
Illustration of gizzard lesions. (**a**) Gizzard of a 11/7127-VT infected bird 10 days post infection (DPI) depicting discoloration, erosion and ablation of the koilin layer. (**b**) Histologically, degeneration and necrosis of the gizzard epithelial cells together with infiltration of inflammatory cells consisting of lymphocytes and heterophils. (**c**) Macroscopic and (**d**) histological presentation of a gizzard without lesions from a bird orally infected with 11/7127-AT at 10 DPI. Haematoxilin and eosin staining. Bar = 100 μm.

Histological lesions in gizzard samples from birds of group 11/7127-VT were observed from 3 DPI onwards. They comprised degeneration of the glandular epithelial cells and loss of koilin layer accompanied by moderate to severe infiltration of macrophages and lymphocytes in the lamina propria, submucosa and muscle layers. At 7 and 10 DPI, numerous inclusion bodies were detected in the glandular epithelial cells of gizzards from birds infected with 11/7127-VT. The majority of the gizzards from group 11/7127-AT showed no histological changes (Figure 4). At 7, 10 and 14 DPI, respectively, in one, two and one out of five birds, an accumulation of lymphocytes was observed in the lamina propria together with slight degeneration of the glandular epithelium. Inclusion bodies were detected sporadically in the gizzard of a single bird at 10 DPI. In group NC, no gross or histopathological lesions were detected.

### 3.4. Virus Excretion and Detection in Target Organs

Table 1 presents a summary of virological results (virus isolation and qPCR) from cloacal swabs and target organs.

**Figure 5:**
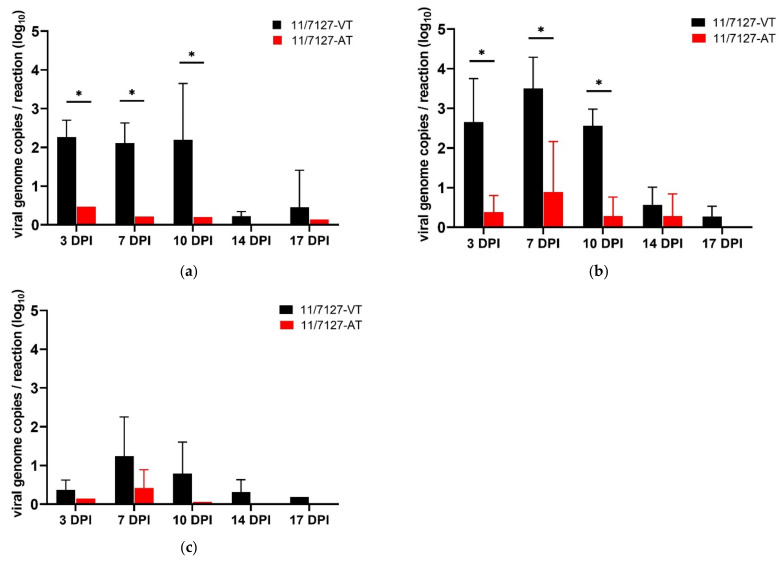
Graphical illustration of viral loads. Mean and standard deviation of viral genome copies per reaction from (**a**) cloacal swab, (**b**) gizzard and (**c**) liver samples calculated by quantitative PCR at 3, 7, 10, 14 and 17 days post infection (DPI) from birds orally infected with 11/7127-VT or 11/7127-AT. Asterisks indicate a significant difference in viral load between group 11/7127-VT and group 11/7127-AT.

In group 11/7127-VT, viable virus was isolated from cloacal swabs until 17 DPI and from gizzard and liver samples until 10 DPI. Throughout the experiment, viral DNA could be detected from 96%, 88% and 80% of the investigated cloacal swab, gizzard and liver samples, respectively. Viral genome copy numbers were determined and mean values for each time point post infection were calculated. In group 11/7127-VT, cloacal swabs showed similar high mean values of viral DNA at 3, 7 and 10 DPI (2.26 ± 0.44, 2.11 ± 0.54 and 2.19 ± 1.46 log_10_ viral genome copies per reaction, respectively); afterwards, viral load decreased. The maximum viral load in gizzard and liver samples was found at 7 DPI (3.50 ± 0.79 and 1.24 ± 1.01 log_10_, respectively) (Figure 5).

In group 11/7127-AT, viable virus was isolated only sporadically from investigated samples. Throughout the trial, viral DNA was recovered by qPCR from 16%, 20% and 40% of the investigated cloacal swab, gizzard and liver samples, respectively. Highest mean values of viral DNA in cloacal swab samples were detected at 3 DPI (0.47 log_10_) and in gizzard and liver samples at 7 DPI (0.89 ± 1.27 and 0.42 ± 0.47 log_10_ viral genome copies per reaction, respectively). In both groups, the highest viral load was detected in gizzard samples. Altogether, samples of group 11/7127-AT contained a lower viral load compared to values found in samples from group 11/7127-VT; significant differences were detected in gizzard and cloacal swab samples from 3 to 10 DPI (*p* < 0.05). Examined by qPCR, all cloacal swabs and organ samples from group NC were negative.

### 3.5. Antibody Development

**Figure 6:**
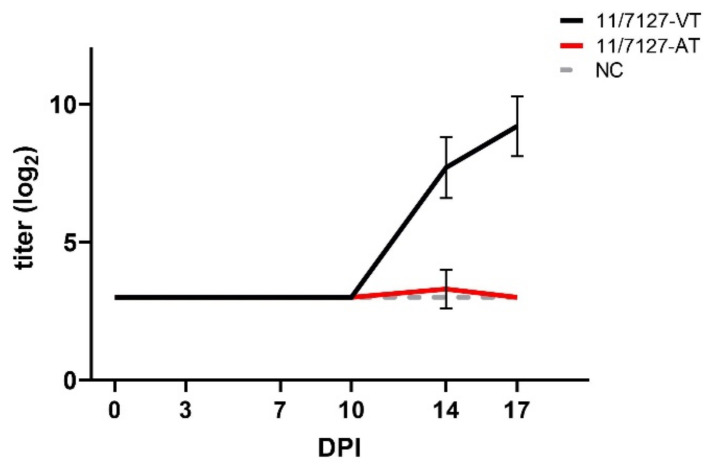
Graphical presentation of virus neutralization test (VNT) results. Mean and standard deviation of FAdV-1 specific VNT titers (log_2_) at 3, 7, 10, 14 and 17 days post infection (DPI) from birds orally infected with 11/7127-VT or 11/7127-AT are shown together with results from the negative control group NC. Titers ≤ 3 log_2_ are considered negative.

In group 11/7127-VT, a homologous antibody response could be detected by VNT in all investigated birds from 14 DPI onwards with mean titers of 7.7 ± 1.1 and 9.1 ± 1.0 log_2_ at 14 and 17 DPI, respectively (Figure 6). Except in one bird showing a titer of 5.0 log_2_ at 14 DPI, no neutralizing antibodies were detected in birds from group 11/7127-AT. Birds of the uninfected group NC did not develop antibodies against FAdV-1 throughout the study.

## 4. Discussion

In the present study, the virulent FAdV-1 strain (11/7127-VT), isolated from 10 day-old broilers during an outbreak of AGE in Germany [13], served as a model to determine the consequences of long-term in vitro passaging in CEL cells on the genome and the virulence of FAdV-1. Previously, we demonstrated the pathogenicity of strain 11/7127-VT in SPF and commercial broilers of different ages [35,36]. In this study, pathogenicity of 11/7127-VT with typical clinicopathological changes was again confirmed after experimental oral infection of day-old SPF layer chicks.

One-step growth kinetics in CEL cells showed notable differences between the investigated strains. After a latent period of 12 h, 11/7127-VT showed rapid exponential virus growth and the corresponding growth curve was very similar to those of previously investigated FAdV strains, independent of the in vitro system (CEL, chicken kidney or chicken hepatoma cells), altogether isolates with limited passages [28,38,40,41]. A similar latent period was observed for the attenuated virus indicating that the length of the replicative cycle was not different per se. However, trends to lower intracellular titers already noticed 6 h post infection indicate some impairment of virus attachment and entry mechanisms; additional investigations 1–2 h post infection would be necessary to confirm this. Despite a slight increase in intracellular virus throughout the investigation, a somewhat flattened growth curve was noticed. Furthermore, release of virus from CEL cells was delayed and a significantly lower virus yield was achieved, indicating decreased replication efficiency of the passaged strain in vitro. Phenotypic characteristics of the FAdV-1 isolate after 53 consecutive passages in CEL cell culture differed significantly from in vitro and in vivo properties of its virulent progenitor strain. Correspondingly, experimental oral infection with 11/7127-AT led to significantly lower detection rates and lower viral loads in gizzard, liver and cloacal swab samples, suggesting decreased replicative and transmission fitness of the passaged and attenuated strain in vivo. Consequently, chicks remained healthy and showed body weight development comparable to the negative control birds. Overall, pathogenicity of strain 11/7127-AT and laboratory analysis were similar to those reported recently for non-pathogenic FAdV-1 reference strains (CELO and OTE), and very much different from pathogenic FAdV-1 [36,42]. Both FAdV-1 reference strains, OTE and CELO, have undergone countless in vitro passages in different substrates since their first description in 1957 and 1964 [36,42]. Previous investigations have suggested that the ability to stimulate an antibody response after oral infection depends on the age of birds as well as the FAdV strain involved [7,23]. Similar to 11/7127-AT, the non-pathogenic FAdV-1 reference strain CELO, also showed reduced production of neutralizing antibodies when given orally to day-old chicks, yet offered protection against adverse effects of a virulent FAdV-1 challenge [36].

In principal, virus pathogenicity and pathogenesis of diseases have been associated repeatedly with viral fitness and the ability of a virus/virus strain to replicate in host organisms, which may be reflected in a high viral load of affected tissues [43]. In the same way, investigations have suggested a straightforward pathogenesis of AGE, with the development of gizzard lesions largely due to the localized FAdV-1 infection and replication in gizzard epithelial cells [35]. Altogether, our observations confirm a very fundamental correlation between viral fitness of individual FAdV-1 strains and the development and progression of AGE.

So far, the majority of studies on molecular differences between virulent FAdV-1 strains and non-virulent FAdV-1 reference strains (CELO and OTE) have focused on only a few genes, whose products fulfill well-known functions in host cell entry [44,45] and in vivo replication and pathogenicity of certain FAdVs [26,46]. Unlike pathogenic FAdV-1 strains from Japan, pathogenic European FAdV-1 isolates could not be distinguished from non-pathogenic strains based on the long fiber sequence [8,9]. Genomic differences in the short fiber knob region were described between pathogenic FAdV-1 strains and CELO but the same mutations were also present in the non-pathogenic OTE strain, contradicting an effect on strain pathogenicity [9]. More recently, whole genome sequence comparisons of both historical and contemporary FAdV-1 isolates including several strains of different pathogenicity, have shown FAdV-A as the most conserved of all FAdV species, with virulence markers in regards of gizzard erosions still unknown [30,47]. The original molecular investigations of hexon, long and short fiber genes grouped 11/7127-VT together with other investigated pathogenic FAdV-1 [9]. However, consistent with the growing awareness that FAdV structural protein sequences cannot be reliably correlated to pathotype differences, those sequences were fully identical between 11/7127-AT and its progenitor genome [30]. The high genomic conservation between FAdV-1 strains also pertains to 11/7127 after long-term consecutive passage on CEL cells. In the genome of our investigated strain, only one sequence length polymorphism as compared to the parental genome was identified, consisting of a two nucleotide addition in a non-coding poly-adenine series immediately distal of the hexon open reading frame. A possible association between this singular genomic change and its altered phenotype should be judged cautiously, particularly because low-complexity regions are overall more prone to be affected by dynamic variations due to the propensity of polymerase slippage in repeat regions, shown in eukaryotes and prokaryotes, but also viruses [48,49]. Consistent with this, poly-adenine motifs of different lengths are also present in the same location in other published FAdV-1 genomes, without an obvious link to the strains’ virulence, although 7127-AT is the only one with the so-far longest repeat expansion. Despite being non-protein coding, the sequence containing the identified mutation is part of the FAdV hexon mRNA 3′ UTR segment [50]. In the absence of any other sequence-based findings to explain differential replication profiles in the investigated strain pair, it, albeit hypothetical, remains the only explanation that alterations in this particular genomic motif play a transcription regulatory role, which is generally described as a common feature of 3′ UTRs [51]. In case of the actual transcription unit, this might also be of interest since the 3′- distal element is the protease, which—if affected temporally or downregulated during transcription—would represent a serious impediment on virus maturation and infection processes.

Finally, terminally encoded products have been suggested receptive to control of host immune components, providing a context for external factors affecting virus infection and replication efficiency [31]. Recently, data demonstrated host cells undergo differential regulation of genes mainly pertaining to the intracellular trafficking machinery (e.g., phagosome pathways) and immune related pathways (e.g., Toll-like receptor, cytokine–cytokine receptor pathways, production of cytokines and chemokines) in response to FAdV infection both in vivo and in vitro [52,53,54,55]. It is thus conceivable, that extracellularly released immune regulatory elements can accumulate during long-term consecutive cultures with a negative impact on viral fitness and consequently pathogenicity of the investigated virus progeny 11/7127-AT.

To our knowledge, this is the first study addressing phenotypic and genomic differences between an in vitro attenuated FAdV-1 and its virulent progenitor strain. Long-term, consecutive passage in CEL cells induced poorer replication fitness in vitro and reduced pathogenicity in day-old SPF chicks, based upon absence of clinicopathological changes after experimental challenge. However, virulence differences of the investigated strain pair could not be attributed to distinct and prominent genomic differences. To elucidate whether observed changes in the poly-adenine tract adjacent to the hexon stop codon play a role in regulation of transcriptional processes with consequences on virulence or whether certain host mechanisms have an effect on the infection cycle of FAdV-1, further investigations are needed.

## Figures and Tables

**Table 1:** Virus isolation and detection of viral DNA. No. of positive / no. of tested gizzard, liver and cloacal swab samples from experimentally infected groups 11/7127-VT and 11/7127-AT at different time points post infection (DPI) are shown. Samples were investigated by virus isolation from chicken embryo liver cell culture (CEL) and by quantitative PCR (qPCR).

	Group 11/7127-VT						Group 11/7127-AT					
	Gizzard		Liver		Cloacal Swabs		Gizzard		Liver		Cloacal Swabs	
	CEL	qPCR	CEL	qPCR	CEL	qPCR	CEL	qPCR	CEL	qPCR	CEL	qPCR
0 DPI	n/a ^a^	n/a	n/a	n/a	0/5	0/5	n/a	n/a	n/a	n/a	0/5	0/5
3 DPI	5/5	5/5	4/5	5/5	5/5	5/5	3/5	3/5	0/5	1/5	0/5	1/5
7 DPI	5/5	5/5	4/5	5/5	5/5	5/5	2/5	3/5	1/5	3/5	1/5	1/5
10 DPI	5/5	5/5	3/5	5/5	5/5	5/5	1/5	1/5	0/5	1/5	1/5	1/5
14 DPI	0/5	4/5	0/5	4/5	3/5	5/5	0/5	3/5	0/5	0/5	0/5	0/5
17 DPI	0/5	3/5	0/5	1/5	2/5	4/5	0/5	0/5	0/5	0/5	1/5	1/5
Total	15/25	22/25	11/25	20/25	20/25	24/25	6/25	10/25	1/25	5/25	3/25	4/25

^a^ n/a: not applicable.

## Data Availability

Publicly available datasets were analyzed in this study. This data can be found here: GenBank under accession number MK572848.

## Supplementary Materials

The following supporting information can be downloaded at: https://www.mdpi.com/article/10.3390/v14020358/s1, Table S1: Primer sequences and description of protocols for Sanger sequencing.
