# Supplementary material for: High Phenotypic Variation between an In Vitro-Passaged Fowl Adenovirus Serotype 1 (FAdV-1) and Its Virulent Progenitor Strain despite Almost Complete Sequence Identity of the Whole Genomes
Source: Viruses. 2022 Feb 9;14(2):358. doi: 10.3390/v14020358 (PMC8880033; doi:10.3390/v14020358)
Supplement: Supplementary file 1 [file viruses-14-00358-s001.zip › viruses-1531973-supplementary.pdf]

Table S1. Primer sequences and description of protocols for Sanger sequencing.

**Primer name**	**Primer sequence (5′ – 3′)**
FAdV1- 37116 fw	GTAGCATACCGGGACAGGT
FAdV1- 37697 rv	CTAATGGTAACGTGGATAATGC
FAdV1- 39835 fw	CAAGTCCGACGCTCTTACG
FAdV1- 40449 rv	GATGTTCCATACCCGGTACT
FAdV1- 40429 fw	CAGTACCGGGTATGGAACAT
FAdV1- 41298 rv	CCAGGATGACGTCATTCATA
FAdV1- 41200 fw	GTACATTCCGGCAAATGTAC
FAdV1- 42174 rv	GGATGACATTTCGGCTATCA
FAdV1- 42114 fw	GGCAATACAAATTTGAATGGCG
FAdV1- 42989 rv	GCAAATGACTGGAATCCAGATC
FAdV1- 42958 fw	AGAATCATCTTAAAGGAGCCCT
FAdV1- 43777 rv	GCACGGTGTCGCTATACG

Oligonucleotides were synthesized at Eurofins MWG Operon (Ebersberg, Germany). All PCR amplifications were carried out in 25 µl reactions containing 3 µl DNA template, 2.5 µl of 10x PCR buffer (Invitrogen, Vienna, Austria), 0.5 µl dNTPs (10 µM stock), 0.75 µl $MgCl_2$ (25 µM stock), 1 µl each primer (25 µM stock), 0.2 µl Taq-Polymerase (1.25 units/mL) and 16.05 µl of ultrapure water. Reactions were run on a Bio-Rad PTC-0220 thermal cycler (Bio-Rad Laboratories, Hercules, CA, USA) using the following parameters: initial denaturation at 94°C for 2 minutes, 35 cycles of 94°C for 1 minute, 52°C for 1 minute and 72°C for 1 minute, followed by a final extension at 72°C for 10 minutes. After electrophoretic separation on a 1% gel for 40 minutes, bands were excised from the gel and DNA extracted with the QIAquick Gel Extraction kit (Qiagen, Vienna, Austria) according to manufacturer's instructions. Sequencing service was carried out by LGC Genomics GmbH (Berlin, Germany).
